# Footprints of Selection Derived From Temporal Heterozygosity Patterns in a Barley Nested Association Mapping Population

**DOI:** 10.3389/fpls.2021.764537

**Published:** 2021-10-14

**Authors:** Andreas Maurer, Klaus Pillen

**Affiliations:** Institute of Agricultural and Nutritional Sciences, Chair of Plant Breeding, Martin Luther University Halle-Wittenberg,Halle, Germany

**Keywords:** barley, heterozygosity, temporal genomic data, adaptive evolution, selection, natural selection, artificial selection, unconscious selection

## Abstract

Nowadays, genetic diversity more than ever represents a key driver of adaptation to climate challenges like drought, heat, and salinity. Therefore, there is a need to replenish the limited elite gene pools with favorable exotic alleles from the wild progenitors of our crops. Nested association mapping (NAM) populations represent one step toward exotic allele evaluation and enrichment of the elite gene pool. We investigated an adaptive selection strategy in the wild barley NAM population HEB-25 based on temporal genomic data by studying the fate of 214,979 SNP loci initially heterozygous in individual BC_1_S_3_ lines after five cycles of selfing and field propagation. We identified several loci exposed to adaptive selection in HEB-25. In total, 48.7% (104,725 SNPs) of initially heterozygous SNP calls in HEB-25 were fixed in BC_1_S_3:8_ generation, either toward the wild allele (19.9%) or the cultivated allele (28.8%). Most fixed SNP loci turned out to represent gene loci involved in domestication and flowering time as well as plant height, for example, *btr1/btr2*, *thresh-1*, *Ppd-H1*, and *sdw1*. Interestingly, also unknown loci were found where the exotic allele was fixed, hinting at potentially useful exotic alleles for plant breeding.

## Introduction

Around 10,000years ago, domestication of crops enabled mankind to settle and to commence agriculture. Year after year, early farmers and breeders selected the best performing plants for the next season. Domestication and selection were accompanied by a progressive depletion of genetic diversity, known as bottleneck effect (Tanksley and McCouch, 1997). To cope with future agricultural challenges, there is a need to replenish the limited elite gene pools with favorable exotic alleles from the wild progenitors of our crops (Zamir, 2008; McCouch et al., 2013; Zhang et al., 2017). However, the identification of beneficial exotic material can be laborious and challenging (Dempewolf et al., 2017).

NAM populations can be developed to investigate a multitude of exotic allele effects in an adapted background. They are created by crossing a diverse set of wild progenitors with one recurrent elite cultivar. This way high allele richness and statistical power are combined to evaluate complex traits through genome-wide association studies (GWAS). Subsequent backcross steps with the elite cultivar may serve as a first step to integrate exotic alleles in adapted breeding material and increase the reliability of estimated wild allele effects. The wild barley NAM population HEB-25 is backcross-based and comprises 1,420 BC_1_S_3_ lines resulting from crosses of 25 highly divergent wild barley accessions (*Hordeum vulgare* ssp. *spontaneum* and ssp. *agriocrithon*) with the elite barley cultivar Barke (Maurer et al., 2015). In this population recombination rate (Dreissig et al., 2020) and several important agronomic traits like plant development (Maurer et al., 2015, 2016; Herzig et al., 2018; Wiegmann et al., 2019a; Pham et al., 2020), yield formation (Maurer et al., 2016; Sharma et al., 2018; Wiegmann et al., 2019a), grain nutrient concentration (Herzig et al., 2019; Wiegmann et al., 2019b), as well as tolerance to abiotic (Saade et al., 2016; Merchuk-Ovnat et al., 2018; Pham et al., 2019) and biotic (Vatter et al., 2017, 2018; Büttner et al., 2020; Mehnaz et al., 2021) stresses were investigated. Although all of these studies proved useful to find genomic regions controlling the investigated traits, in many cases, it remains unclear which effects can truly be designated as beneficial across certain environments. For instance, a clear statement about the usefulness of flowering time affecting wild alleles is often not possible due to their environment-dependent phenotypic plasticity (Guo et al., 2020; Li et al., 2021), complicating the transfer of beneficial alleles from one environment to another.

In this context, outsourcing the job of selection to mother nature may help to define what is beneficial in a certain environment. Over the long term, this enables the prevalence of certain ideotypes with a complex of optimally coordinated properties rather than focusing on single traits in classical selection. This principle of natural selection is key of the evolutionary plant breeding concept (Suneson, 1956; Phillips and Wolfe, 2005; Döring et al., 2011).

In the present study, we investigated an adaptive selection strategy in HEB-25 by temporal screening of initially heterozygous loci (where 6.25% of the genome is expected to be heterozygous in each HEB line in generation BC_1_S_3_) after 5years of selfing and field propagation without conscious selection as a by-product of population conservation. A clear fixation of exotic alleles could hint at potentially useful exotic alleles that were more successful in contributing to the next field generation.

## Materials and Methods

### Plant Material

The wild barley nested association mapping (NAM) population HEB-25 resulted from parallel crosses of 25 highly divergent wild barley accessions (*Hordeum vulgare* ssp. *spontaneum* and ssp. agriocrithon, hereafter named *Hsp*) with the German elite barley cultivar Barke (*Hordeum vulgare* ssp. *vulgare*, hereafter named *Hv*). F1 plants of the initial crosses were backcrossed with Barke and after three subsequent rounds of selfing the population comprised 1,420 BC_1_S_3_ plants (Maurer et al., 2015). Due to the mating design, each BC_1_S_3_ plant is expected to harbor 71.875% homozygous Barke loci, 6.25% heterozygous loci, and 21.875% homozygous wild loci under the assumption of no selection.

### Population Conservation

**Figure 1:**
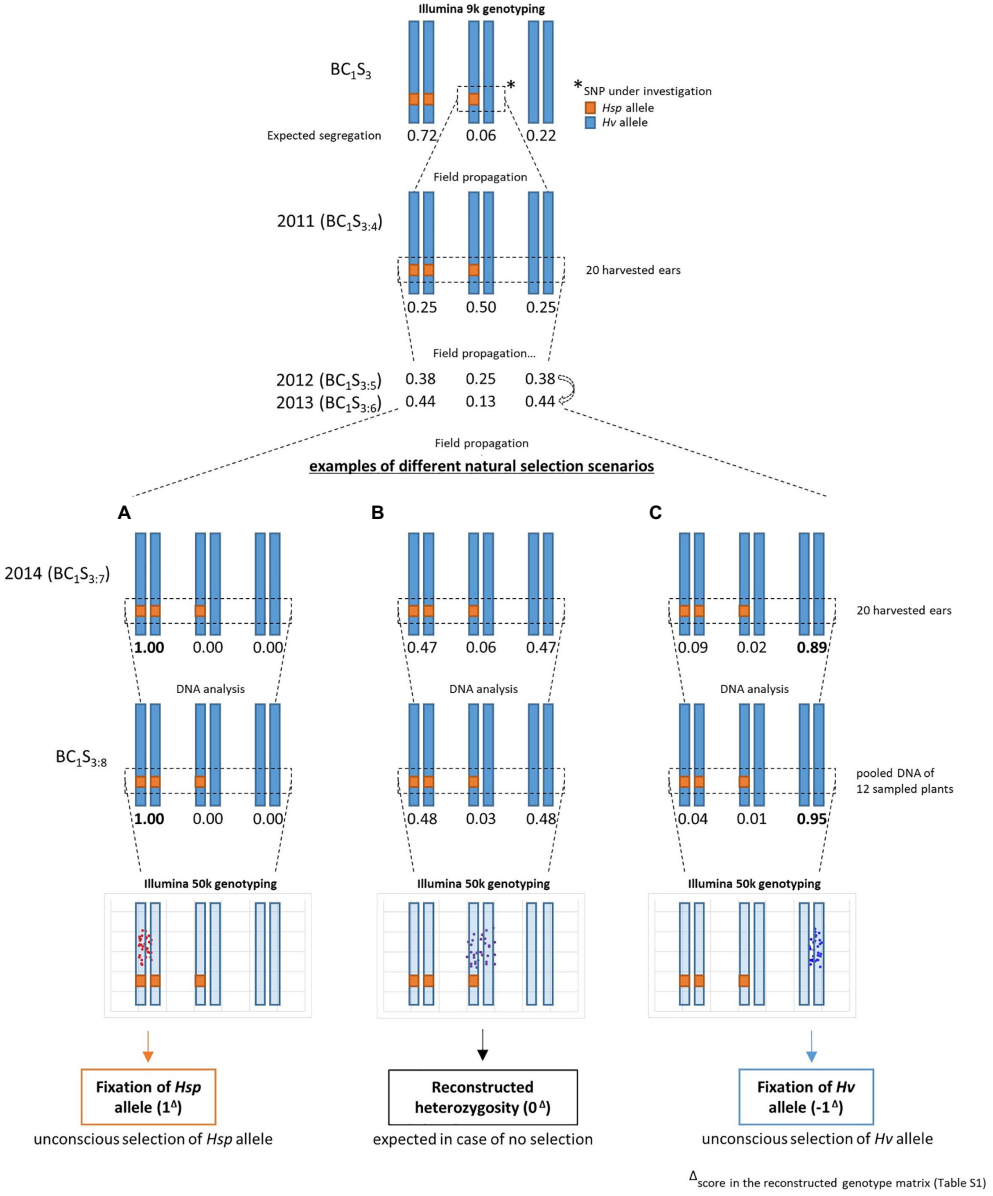
Population conservation and sampling strategy from generation BC_1_S_3_ to BC_1_S_3:8_. Exemplified for one heterozygous SNP in BC_1_S_3_, which is traced through four seasons of field propagation (2011–2014) followed by pooled DNA sampling of 12 seedlings in BC_1_S_3:8_. Heterogeneity within these 12 seedlings leads to heterozygous allele calls in Illumina 50k genotyping and is termed “reconstructed heterozygosity”. The expected segregation of a single SNP in HEB-25 generation BC_1_S_3_ is equal to 71.875% homozygous Barke, 6.25% heterozygous, and 21.875% homozygous wild barley. Without selection, the expected SNP segregation of the offspring of a heterozygous HEB-25 plant in generation BC_1_S_3:8_ is equal to 48.4375% homozygous Barke, 3.125% heterozygous, and 48.4375% homozygous wild barley, giving rise to a reconstructed heterozygous genotype **B**, resulting in a score of 0 in the reconstructed genotype matrix; Supplementary Table S1). **A** and **C** indicate different possible scenarios of allele distribution in case of selection for *Hsp* allele **A**, resulting in a score of 1 in the reconstructed genotype matrix) or *Hv* allele (**C**, resulting in a score of −1 in the reconstructed genotype matrix) in previous generations (here exemplified for BC_1_S_3:7_). Note that a homozygously fixed allele call in Illumina genotyping can be obtained despite the presence of small amounts of opposite alleles in the pooled sample **C**.

In 2011, BC_1_S_3_-derived lines were created by growing the complete progeny of each single BC_1_S_3_ plant (i.e., BC_1_S_3:4_) in small plots (double rows of 1.50m length) in the field (Halle, Germany, 51°29′46.47″N; 11°59′41.81″E) and 20 randomly chosen ears of each line, displaying a representative subset of the whole plot, were harvested at maturity. After threshing and manual seed processing, 60 seeds thereof were randomly selected for sowing in 2012 (i.e., BC_1_S_3:5_). The same process of harvesting, processing, and sowing was repeated with 60 BC_1_S_3:6_ seeds in 2013 and 100 BC_1_S_3:7_ seeds in 2014. Then, 20 BC_1_S_3:8_ seeds (harvested from the BC_1_S_3:7_ plants in 2014) were grown and leaf material from 12 randomly chosen seedlings per line (50–100mg) was harvested to form pooled samples for DNA extraction. This way, initial heterozygosity could be reconstructed through heterogeneity within the 12 plants (Figure 1).

### DNA Extraction and SNP Genotyping

DNA was extracted according to the manufacturer’s protocol, using the BioSprint 96 DNA Plant Kit and a BioSprint96 work station (Qiagen, Hilden, Germany), and finally dissolved in distilled water at approximately 50ng/μl. The original 1,420 BC_1_S_3_ plants were genotyped with the barley 9k Infinium iSelect SNP array (Maurer et al., 2015), consisting of 7,864 SNP markers as reported in Comadran et al. (2012). Pooled DNA samples of 12 BC_1_S_3:8_ seedlings per HEB-25 line were genotyped with the barley 50k Infinium iSelect SNP array (Bayer et al., 2017; Maurer and Pillen, 2019) at TraitGenetics GmbH, Gatersleben, Germany to reconstruct original heterozygosity. SNP calling in Illumina genotyping assays is based on the concept of hybridization technology with specifically designed oligonucleotide probes, where the intensity of two distinct fluorescently labeled target sequences represents the signal strength for each of the two alleles. Then, a cluster algorithm is applied to distinguish the two contrasting homozygous classes and heterozygous calls (Zhao et al., 2018). At TraitGenetics GmbH, the cluster files determining the thresholds for allelic discrimination have been manually revised to improve the call quality, both for the 9k and the 50k array. After SNP calling, SNP markers that did not meet the quality criteria (polymorphic in at least one HEB family, < 10% failure (i.e., no call) rate, and<12.5% heterozygous calls, which is twice the expectancy in BC_1_S_3_) were removed from the dataset. Furthermore, 256 SNPs were removed as they revealed exact segregation among all HEB lines, indicating that they were in complete linkage disequilibrium (LD). Only one of these duplicates was kept. Altogether, 4,717 SNPs, genotyped in both SNP arrays, met the quality criteria. In the present study, 57 of the initial 1,420 lines were eliminated due to clearly inconsistent genotypes between BC_1_S_3_ and BC_1_S_3:8_.

### Determination of Allele Fixation at Originally Heterozygous SNPs

Only heterozygous loci in BC_1_S_3_ generation were screened for their allele state in BC_1_S_3:8_. The allele fixation rate (AFR) for each SNP was determined as


$$AFR_{SNP}=\frac{\begin{array}{l} \mathrm{Number of homozygous calls across}\;\mathrm{all}\;\mathrm{initially}\\ \mathrm{heterozygous}\;\mathrm{HEB}\;\mathrm{lines in}\;BC_{1}S_{3:8} \end{array}}{\begin{array}{l} \mathrm{Number of heterozygous calls across}\;\\ \mathrm{all}\;\mathrm{HEB}\;\mathrm{lines in}\;BC_{1}S_{3} \end{array}}.$$

To determine the relative fixation direction (RFD) in which an originally heterozygous SNP allele moved, a reconstructed genotype matrix was created (Supplementary Table S1) containing fixation values for each SNP*genotype combination where “0” represents SNPs that retained their (reconstructed) heterozygosity state, i.e., both alleles are still present in the 12 sampled plants of a BC_1_S_3:8_ line, “-1” represents SNPs that were fixed for the homozygous *Hv* allele, and “+1” represents SNPs that were fixed for the homozygous *Hsp* allele. The RFD for each SNP was then determined as


$$RFD_{SNP}=\frac{\begin{array}{l} \mathrm{Sum}\;\mathrm{of fixation values}\;\left(i\mathrm{.e}.-1,0,1\right)\;\mathrm{across}\;\mathrm{all}\;\\ \mathrm{initially heterozygous}\;\mathrm{HEB}\;\mathrm{lines in}\;BC_{1}S_{3:8} \end{array}}{\begin{array}{l} \mathrm{Number of heterozygous calls across}\;\\ \mathrm{all}\;\mathrm{HEB}\;\mathrm{lines in}\;BC_{1}S_{3} \end{array}}.$$

Both AFR and RFD were only calculated for SNPs containing at least 10 heterozygous HEB lines in generation BC_1_S_3_ (n=3,872 SNPs) to avoid strong bias.

To test for significance of RFD, chi-square goodness-of-fit tests were conducted to measure deviations from a 1:1 ratio of homozygous *Hv* to homozygous *Hsp* genotypes in BC_1_S_3:8_. For this purpose, a sliding window approach summarizing allele counts of 10 consecutive SNPs was applied. A significant deviation was accepted at a Bonferroni-corrected value of *p* < 0.01.

Pearson’s correlations of RFD with *Hsp* allele SNP effects estimated in the whole HEB-25 population were calculated. For this purpose, SNP effects were obtained from a simple linear model regressing published data on plant height and flowering time (Maurer et al., 2016), ear number, grain number per ear, grain yield, and threshability (Herzig et al., 2019), as well as unpublished data of powdery mildew susceptibility and brittleness of rachis on the quantitative SNP scores (matrix D of Maurer and Pillen (2019)).

### Expected Probabilities of Allele Fixation During Population Conservation

The expected segregation ratio in the BC_1_S_3_ generation of HEB-25 is 0.71875: 0.0625: 0.21875 for homozygous *Hv* allele, heterozygous, and homozygous *Hsp* allele, respectively. Those 6.25% of heterozygous SNPs will segregate in advanced generations. In each selfing generation, the heterozygous rate is halved and a quarter is going to be fixed in each of the two homozygous classes. The progeny of a single BC_1_S_3_ plant, which is termed BC_1_S_3:4_, is therefore expected to segregate in a 0.25: 0.5: 0.25 ratio at an initially heterozygous SNP. By following this rule, a segregation of 0.484375: 0.03125: 0.484375 at initially heterozygous loci is expected for BC_1_S_3:8_ generation, if no selection occurs during reproduction and plant cultivation (Figure 1B). Consequently, at an initially heterozygous locus, an unbiased reconstructed genotype in BC_1_S_3:8_ should consist of a pooled DNA sample of ~6 *Hv* and~6 *Hsp* plants, giving rise to a heterozygous allele call in 50k genotyping. This way, the initial heterozygosity can be reconstructed. In case of no selection, 100% heterozygous calls are expected in the pooled sample of 12 BC_1_S_3:8_ plants originating from an initially heterozygous BC_1_S_3_ plant.

To estimate the impact of accidentally biased sampling on genotype calling in BC_1_S_3:8_ generation, the probabilities of obtaining a biased pooled sample of 12 plants, used for 50k genotyping, were determined in 1,000,000 binomial trials with regard to different simulated allele segregations (0.05–0.95) of the previously harvested generation. The probabilities of accidental sampling of 12 or≥9 plants of the same homozygous allele class, leading to homozygous genotype calls in Illumina genotyping, were then estimated for each preset allele segregation.

## Results

**Figure 2:**
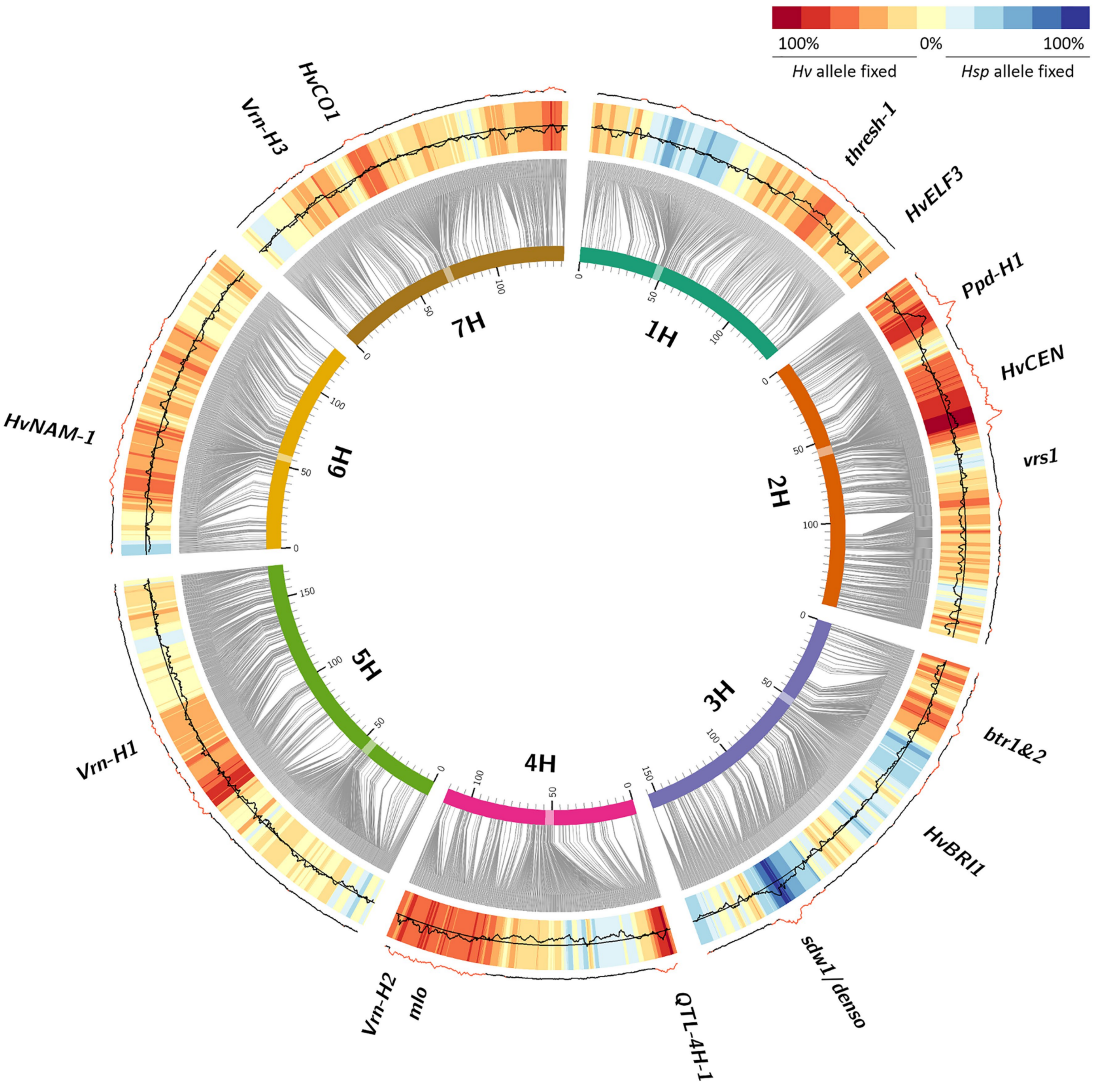
Allele fixation from BC_1_S_3_ to BC_1_S_3:8_ generations in HEB-25. Barley chromosomes are indicated as inner circle of colored bars; centromeres are highlighted as transparent boxes. Grey connector lines represent the genetic position (in cM) of SNPs on chromosomes based on Maurer et al. (2015). The black line indicates the allele fixation rate (AFR) of SNPs in BC_1_S_3:8_ calculated from originally heterozygous HEB-25 lines, summarized over 10 consecutive SNPs. The central reference line indicates the average AFR of 0.486, while the limits of the heat map represent AFRs of 0 (inner) and 1 (outer). AFRs higher than 0.5 represent locus-specific above-average fixation throughout the whole HEB-25 population in BC_1_S_3:8_. The heat map represents the relative fixation direction (RFD), indicating the tendency which allele was fixed in originally heterozygous HEB-25 lines, summarized over 10 consecutive SNPs. Red regions in the outer line indicate significant deviations of RFD from an equal segregation at *P*(Bonferroni)<0.01. Candidate genes of barley domestication and flowering time as well as further striking genomic regions are indicated outside.

In total, 214,979 (6,02%) heterozygous SNP calls were obtained from 3,573,466 polymorphic SNP assays in generation BC_1_S_3_ of population HEB-25 (Supplementary Table S1), which is close to the expected frequency of 6.25%. This represents an average number of≈46 heterozygous HEB lines per SNP. Altogether, 104,725 (48.7%) SNP calls out of those initially heterozygous SNPs were homozygously fixed after five cycles of selfing in BC_1_S_3:8_ generation, based on SNP analysis of a pooled sample of 12 plants for each BC_1_S_3:8_ line. This is also visible in the frequency distribution of the allele fixation rate (AFR) of each single SNP (Supplementary Figure S1; Supplementary Table S1), indicating that on average originally heterozygous SNPs did not segregate equally into both allele classes in half of the lines (average AFR=48.6%). In 42,752 (19.9%) cases, the SNP was fixed toward the *Hsp* allele, while in 61,973 (28.8%) cases, the SNP was fixed toward the *Hv* allele (Supplementary Table S1). Mapping these tendencies on the genome revealed clear patterns of genomic regions where the *Hsp* allele and the *Hv* allele were favored, respectively (Figure 2), enabling to separate the observed effects from genetic drift, which would not favor a specific allele and therefore would result in light yellow colors on the heat map. We expected that Barke (Hv), as a semi-dwarf European spring barley cultivar with resistance to powdery mildew, should be well adapted to the environmental conditions during propagation in the spring-sown trials in Halle. The obviously relevant loci *Ppd-H1*, *Vrn-H2*, mlo, and sdw1/denso were all under selection (Figure 2). AFR was highest for *Ppd-H1* (>90%) and showed a clear tendency of fixation for the Hv allele (negative RFD). This was also true for the Vrn-H2 and mlo loci conferring vernalization independency and resistance to powdery mildew, respectively. However, at denso/sdw1, the Hsp allele was clearly favored (positive RFD).

## Discussion

### Technical Factors Affecting Allele Fixation

One challenge of conserving extensive experimental plant populations with remaining heterozygosity is to develop a strategy to maintain the population in an effective manner. We applied a method of maintaining the barley NAM population HEB-25 by harvesting 20 randomly selected representative ears from each of the 1,420 lines during four seasons of field propagation. By comparing genotype data of the original BC_1_S_3_ plants with a pooled DNA sample of the resulting progeny in BC_1_S_3:8_, we observed that the original heterozygosity present in the HEB lines was halved as indicated by the reconstructed heterozygosity in BC_1_S_3:8_. In other words, half of the lines segregating for a specific SNP locus were fixed toward a homozygous allele during population conservation. To interpret this number, one has to consider that the obtained genotype score is the product of leaf sampling for DNA extraction and subsequent microarray-based SNP genotyping and allele calling. Since the initial heterozygosity declines after five selfing generations, we collected leaf material of 12 plants to reconstruct heterozygosity. Noteworthy, the selection of 12 plants itself harbors the potential to bias the true heterozygosity score if by chance only plants with the same homozygous genotype are collected. However, the probability that either only *Hv* or only *Hsp* genotypes are collected in this way is <0.2% for each homozygous allele class (Supplementary Table S2), assuming that both alleles occur in an equal expected proportion of 0.484375 in BC_1_S_3:8_ (Supplementary Figure S2). In microarray-based SNP genotyping, allele classes are defined with a certain tolerance for the fluorescence signals of both tested nucleotides. This means that a homozygous genotype call can be obtained even if there are up to ~20% of the opposite allele in the pooled DNA sample. If taking this into account, the probability of allele fixation is technically raised, though still rather low (<6% for each homozygous allele class; Supplementary Table S2). The observed AFR of ~0.5 could only be realized if the fractions of harvested seeds from the previous generation(s) are skewed (Supplementary Figure S2), either by natural or artificial selection. It must be noted that in addition to the previous remarks also the fact matters that the cluster file for allele discrimination was revised for the 50k array, possibly leading to a *per se* reduced rate of heterozygous calls. This can explain the relatively high AFR of ~0.5 throughout the whole genome without indication of a specifically selected locus.

### Sources of Selection Pressure During Population Conservation

Deviations from this background noise may hint on a certain selective pressure. During field propagation of a population, many natural sources of selection pressure may exist. For instance, under extreme environmental conditions, specific genotypes may be lost if they cannot cope with existing selection pressure. Furthermore, plant–plant competition occurs in a plot, leading to an unequal contribution of alleles to subsequent generations. Lower sowing densities could help to mitigate this competition to maintain the genotype during plot propagation. However, also artificial selection can occur during maintaining a population, for instance by unconsciously harvesting the most vital looking ears or by chance harvesting unequal fractions of ears of both genotype classes. Therefore, for future studies, we recommend harvesting whole plots rather than a sample of each plot to avoid a potential source of artificial selection. In our case, we assume that a mixture of both sources of selection acted on HEB-25 lines during field cultivation.

### Genomic Regions Under Selection

**Figure 3:**
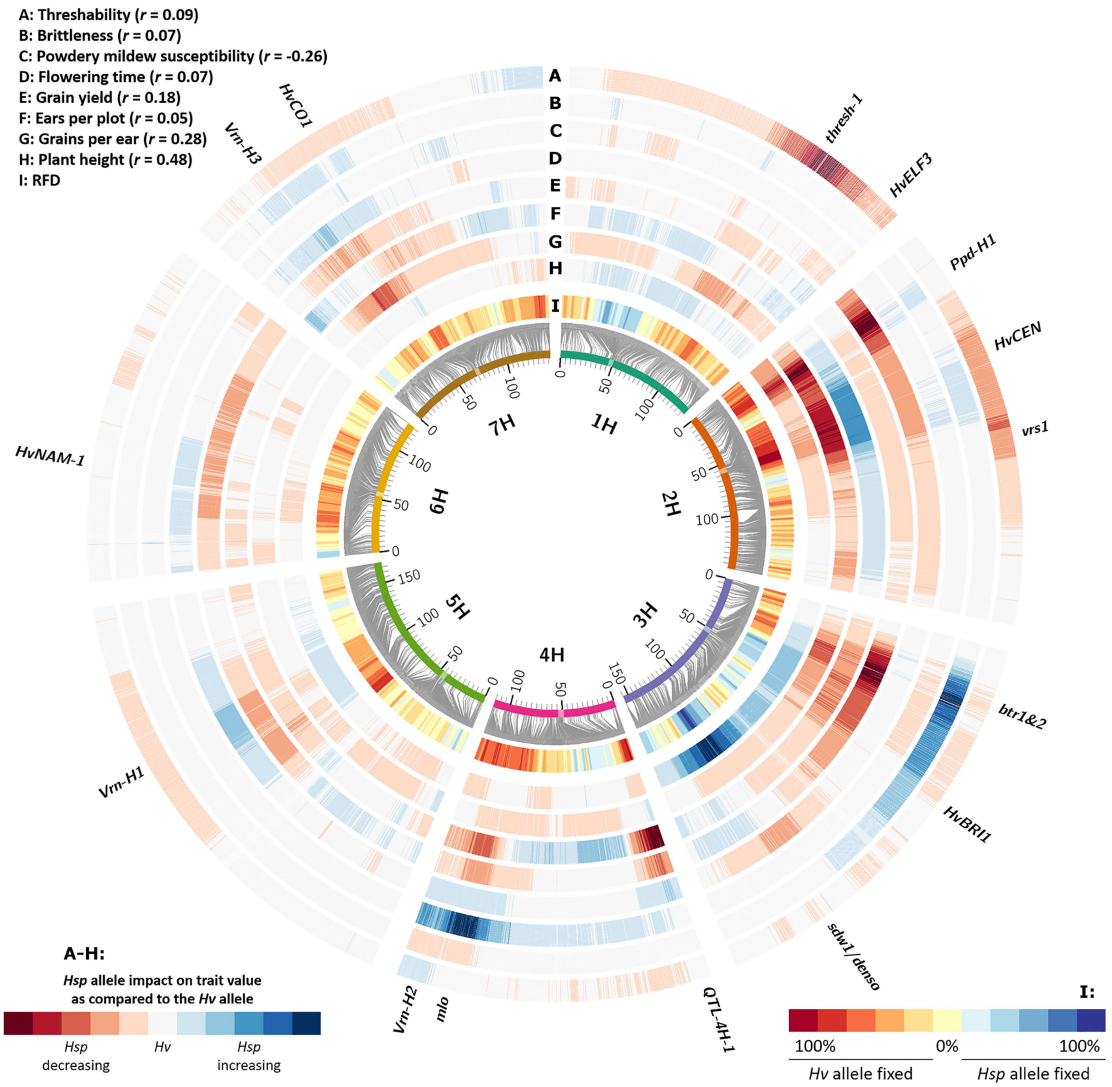
Relative fixation direction (RFD) compared to *Hsp* allele effects of different agronomic traits. Barley chromosomes are indicated as inner circle of colored bars; centromeres are highlighted as transparent boxes. Grey connector lines represent the genetic position (in cM) of SNPs on chromosomes based on Maurer et al. (2015). The inner heat map **(I)** represents the RFD, indicating the tendency which allele was fixed in originally heterozygous HEB-25 lines, summarized over 10 consecutive SNPs. The other heat maps **(A-H)** indicate *Hsp* allele effect size and direction as compared to the *Hv* allele, based on linear regression. The extreme values of the heat maps were standardized to the most extreme observed absolute *Hsp* allele effect for each trait. Candidate genes of barley domestication and flowering time as well as further striking genomic regions are indicated outside.

Interestingly, we observed a systematic pattern of genomic regions being fixed either toward *Hv* or *Hsp* alleles (Figure 2; Supplementary Table S1). Those genomic regions are likely the reasons for selection events that occurred during population conservation. Assigning candidate genes to these regions revealed that most of the loci correspond to well-known genes of barley domestication. Since HEB-25 results from crosses of wild barley and the domesticated elite barley cultivar Barke, this finding can be interpreted as a short story of domestication in barley. Supporting this, there was a clear tendency of selection against the dominant *Btr1/Btr2* allele on chromosome 3H (Pourkheirandish et al., 2015) conferring a brittle rachis and shattering of the ear of wild barley at maturity. In BC_1_S_3:8_ lines, the genomic region of *btr1/btr2* was predominantly fixed toward the cultivated *Hv* allele. Plausibly, predominately intact ears without brittle rachis were harvested, leading to a fixation of non-brittle ears in future generations. This is rather a source of artificial selection due to harvesting than a natural advantage of non-brittle lines. Likewise, another potentially artificially selected genomic region is the threshability locus *thresh-1* on 1H (Schmalenbach et al., 2011). Here, the wild allele causes awns and rachis remaining attached to the grain after mechanical threshing. In contrast, the domesticated dominant allele confers a “clean” grain that can directly be used for sowing in the next season. Although for all grains remaining awns and rachis were manually removed, there might have been the tendency that already “clean” seeds were preferred for next season sowing. Another prominent domestication locus, *vrs1*, leading to the six-rowed ear phenotype in cultivated barley (Komatsuda et al., 2007), could not reliably be captured in our study. We would expect a clear shift toward the six-rowed phenotypes, since the probability that their grains contribute to the next generation is increased due to the higher number of grains per ear. For the sake of completeness, however, it should be mentioned that this effect could be partly compensated by the increased seedling vigor of larger seeds produced in two-rowed ears (Boyd et al., 1971). However, only a single HEB family (F24) shows six-rowed ears, derived from its *H. v*. ssp. *agriocrithon* parent HID380. Only one out of 56 lines of F24 was heterozygous at the *vrs1* locus (BOPA2_12_30897) in BC_1_S_3_. Therefore, a clear statement for this locus is not possible. However, in general, we observed that loci affecting the grain number per ear in HEB-25 (Herzig et al., 2019) were often associated with genomic regions with an increased AFR. This may indicate that more harvested grains from genotypes carrying a grain number-increasing locus lead to a higher probability of those grains being selected for next season sowing. This tendency is supported by the slightly positive correlation of 0.28 between *Hsp* allele SNP effects for grain number and RFD (Figure 3; Supplementary Figure S3).

### Phenology-Related Effects

**Figure 4:**
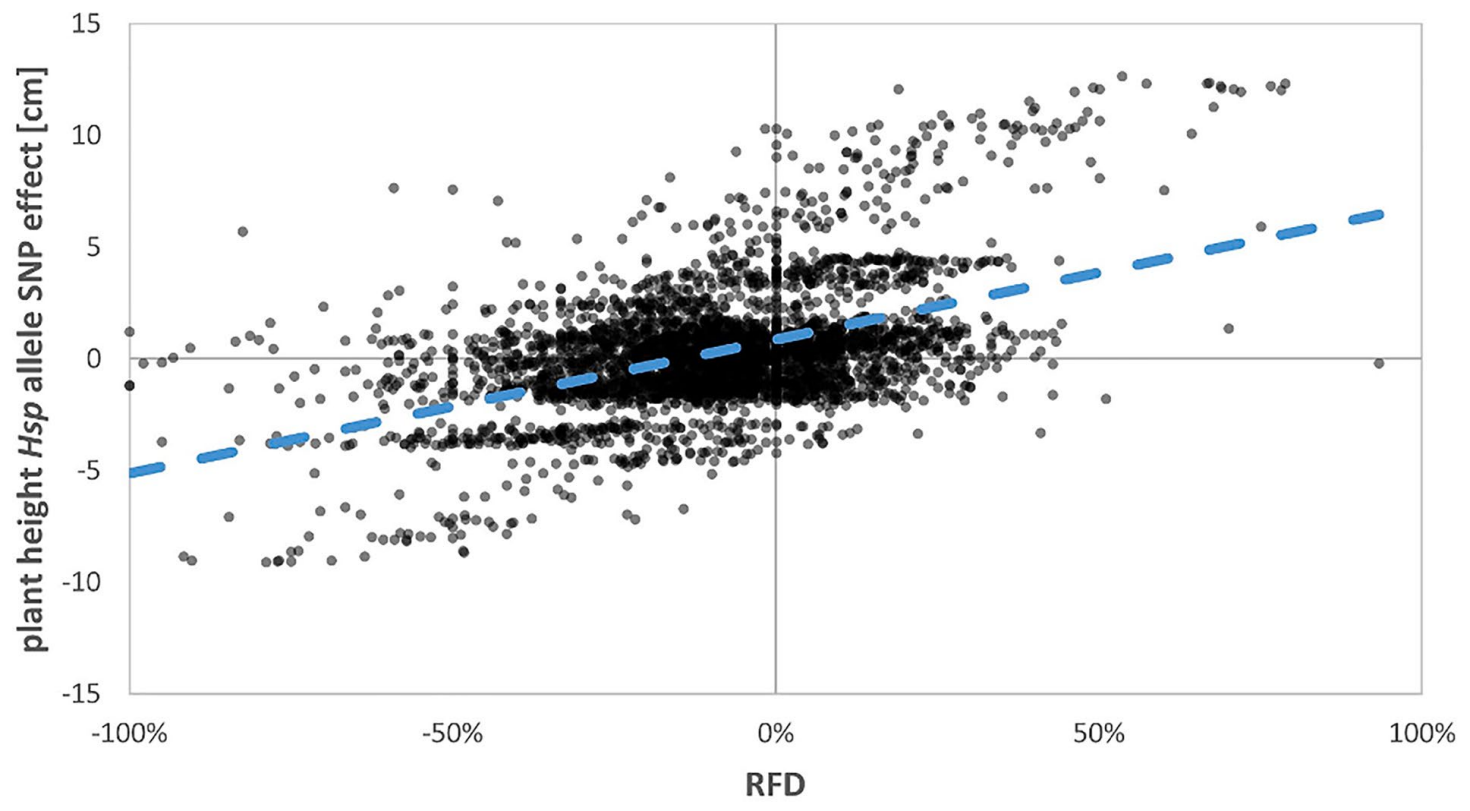
Regression of estimated *Hsp* allele SNP effect for plant height on RFD. The Pearson correlation coefficient (*r*) is 0.48. The blue dashed line indicates the linear regression line.

Strikingly, many of the selection-affected regions correspond to flowering time and plant development genes (Figure 2; Supplementary Table S1). As indicated in Maurer et al. (2015) and Maurer et al. (2016), eight major flowering time loci could be identified in HEB-25 that explained large proportions of the variance for flowering time and other developmental traits. Interestingly, most of them showed a clear fixation tendency. However, the direction of fixation differed and was not correlated with the accelerating or delaying effects of the *Hsp* alleles (Figure 3; Supplementary Figure S4). At these loci predominately, the *Hv* allele was fixed, except at the *sdw1* locus, where the *Hsp* allele was fixed more often. This finding indicates that the reason for the fixation might be a selection for higher plants rather than flowering time, since *sdw1* is the main determinant of plant height in HEB-25 and Barke carries the semi-dwarf allele. Either higher plants were favored at manual harvesting avoiding strenuous stooping or higher plants impeded growth of semi-dwarf plants early on. The latter is supported by Raggi et al. (2016), who also detected several plant height associated genetic loci under natural selection in machine-harvested trials with barley composite crosses, and by Chen et al. (2018), who substantiated that taller plants were more competitive in terms of light interception. Most of the obtained fixed loci in the present study co-localize with QTL for plant height in HEB-25 (Maurer et al., 2016), which was underlined by a correlation of *r*=0.48 between *Hsp* allele effects for plant height at maturity and RFD (Figures 3, 4), indicating that alleles increasing plant height were preferentially fixed. Most likely, those alleles increase competitiveness already early on during plant development. In spring wheat, cultivars with increased plant height showed an increased weed suppression ability (Murphy et al., 2008). Conscious selection on early competitiveness might be useful to breed new cultivars with increased weed suppression ability enabling a sustainable herbicide-reduced cropping system.

One of the prerequisites for the expansion of barley cultivation during domestication was the development of a spring growth habit. In contrast to wild barley, spring barley has no or reduced vernalization requirement and is insensitive to day length allowing for an extended vegetative growth period under long-day conditions (Pourkheirandish and Komatsuda, 2007). The optimum exploitation of the growth period results in higher grain yield and is, therefore, most likely the reason why major flowering time genes were fixed toward the *Hv* allele, which also indicates that Barke (*Hv*) seems to be phenologically optimally adapted to the investigated environment Halle. The most extreme example in this context is *Ppd-H1*, the main determinant of photoperiod response in barley (Turner et al., 2005). Out of the 78 heterozygous lines in BC_1_S_3_ 74 (95%) were fixed in BC_1_S_3:8_ (Supplementary Table S1, BK_12). Thereof, 67 lines were fixed toward the *Hv* allele conferring photoperiod insensitivity. This is among the highest AFR observed throughout the whole genome and outlines the importance of *Ppd-H1* for adaptation to Central and Northern European climates. However, interestingly, in 7 lines, the *Hsp* allele of *Ppd-H1* was fixed in BC_1_S_3:8_. We assume that this indicates random selection events or is due to simultaneous co-selection for another locus, as we do not see any indication of a specific *Ppd-H1* haplotype being preferred (Maurer et al., 2015). Note that the observed fixation of *Ppd-H1* is environment-specific and would probably be less pronounced or even opposite in other latitudes. For instance, in the Mediterranean region, earliness is key to escape early season terminal drought, which would mean a selection advantage for the *Hsp* allele (Andrés and Coupland, 2012; Wiegmann et al., 2019a; Fernández-Calleja et al., 2021).

### Further Interesting Insights

Besides many known domestication loci, also other genomic regions showed specific allele preferences, although the AFR was not higher than the background noise in the rest of the genome. However, the clearly directed fixation at these loci might point to the fixation of alleles in specific families of the NAM population. Examples are the centromere regions of chromosomes 1H and 3H, where a significant tendency toward the *Hsp* allele was observed. This was also true for distal parts of chromosome 3HL and 6HS. All these regions might harbor wild barley alleles that create a positive selection pressure, hinting to promising sources of new allelic variation for future breeding. These wild barley alleles may confer (a)biotic stress tolerance, for instance against drought, which frequently occurs in the studied environment Halle. In 2011, the first year of field propagation, the lowest total precipitation sum until maturity was observed (Supplementary Figure S5). However, one has to admit that these loci might also be a source of artificial selection.

In contrast to looking for an increased AFR, also the opposite approach is interesting. Loci with a low fixation rate might hint to alleles conferring hybrid vigor and might represent potential candidates for hybrid barley breeding. In this context, the peri-centromeric region of chromosome 4H and a region on the long arm of chromosome 2H might be promising targets.

## Conclusion

By screening heterozygosity patterns in a wild × elite barley NAM population, we were able to determine loci affected by selective fixation of either the exotic or the elite barley allele during five cycles of reproduction and field cultivation. The factors causing allele frequency changes through competition in composite crosses are manifold (Blijenburg and Sneep, 1975; Enjalbert et al., 1999; Steele et al., 2004; Phillips and Wolfe, 2005; Raggi et al., 2016). The selection factors may be grouped into (i) natural selection caused by plant–plant competitiveness, phenological advantages as well as superior abiotic and biotic stress resilience and (ii) artificial selection through cultivation and harvesting practices. With our approach, we could unveil the genetic basis of those selection events and define alleles which are superior during reproduction and plant cultivation in the investigated environment. The use of a large segregating mapping population for the temporal screening of heterozygosity enabled to define alleles in distinct genome regions as drivers of adaptive evolution. Defining such superior alleles could help to select chromosomal regions covering potentially beneficial wild alleles conferring, for example, stress tolerance, which is of special importance to cope with drastic upcoming challenges in times of climate change.

## Data Availability Statement

The original contributions presented in the study are included in the article/Supplementary Material, further inquiries can be directed to the corresponding author. Original genotype matrices that were used for the analysis are available in Maurer et al., (2015) and Maurer and Pillen (2019). The resulting fixation matrix is available in Supplementary Table S1.

## Author Contributions

AM analyzed data and wrote the manuscript. KP coordinated the project and co-wrote the paper. All authors contributed to the article and approved the submitted version.

## Funding

This work was supported by the German Research Foundation (DFG) *via* priority program 1530: Flowering time control – from natural variation to crop improvement (grant Pi339/7–1) and *via* the European Research Area Network for Coordinating Action in Plant Sciences (ERA-CAPS, grant Pi339/8–1). Open access publishing was funded by the publication fund of Martin Luther University Halle-Wittenberg.

## Conflict of Interest

The authors declare that the research was conducted in the absence of any commercial or financial relationships that could be construed as a potential conflict of interest.The handling editor declared a shared consortium with one of the authors KP at time of review.

## Publisher’s Note

All claims expressed in this article are solely those of the authors and do not necessarily represent those of their affiliated organizations, or those of the publisher, the editors and the reviewers. Any product that may be evaluated in this article, or claim that may be made by its manufacturer, is not guaranteed or endorsed by the publisher.
