# Supplementary material for: Footprints of Selection Derived From Temporal Heterozygosity Patterns in a Barley Nested Association Mapping Population
Source: Front Plant Sci. 2021 Oct 14;12:764537. doi: 10.3389/fpls.2021.764537 (PMC8551860; doi:10.3389/fpls.2021.764537)
Supplement: Supplementary file 1 [file Data_Sheet_1.pdf]

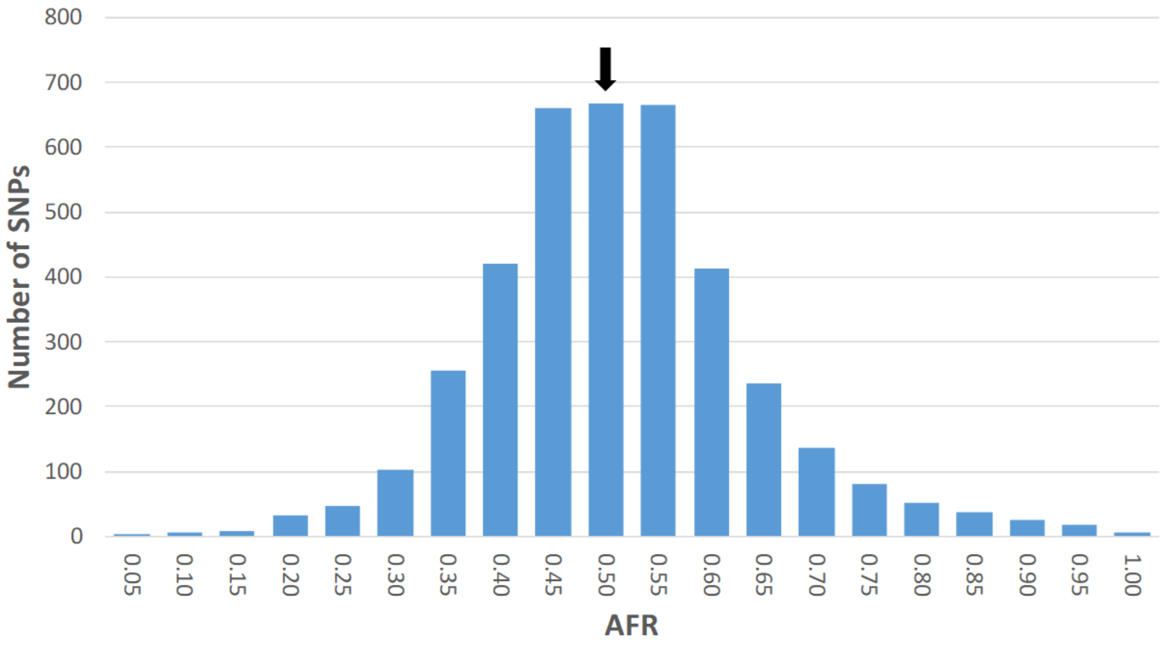

Figure S1. Histogram of the allele fixation rate (AFR) of initially heterozygous SNPs in HEB-25. AFR describes the relative amount of homozygous fixed HEB-25 lines in $BC_1S_{3:8}$ generation derived from heterozygous HEB-25 lines in BC_1S_3. The black arrow indicates the class of the average AFR (=0.486).

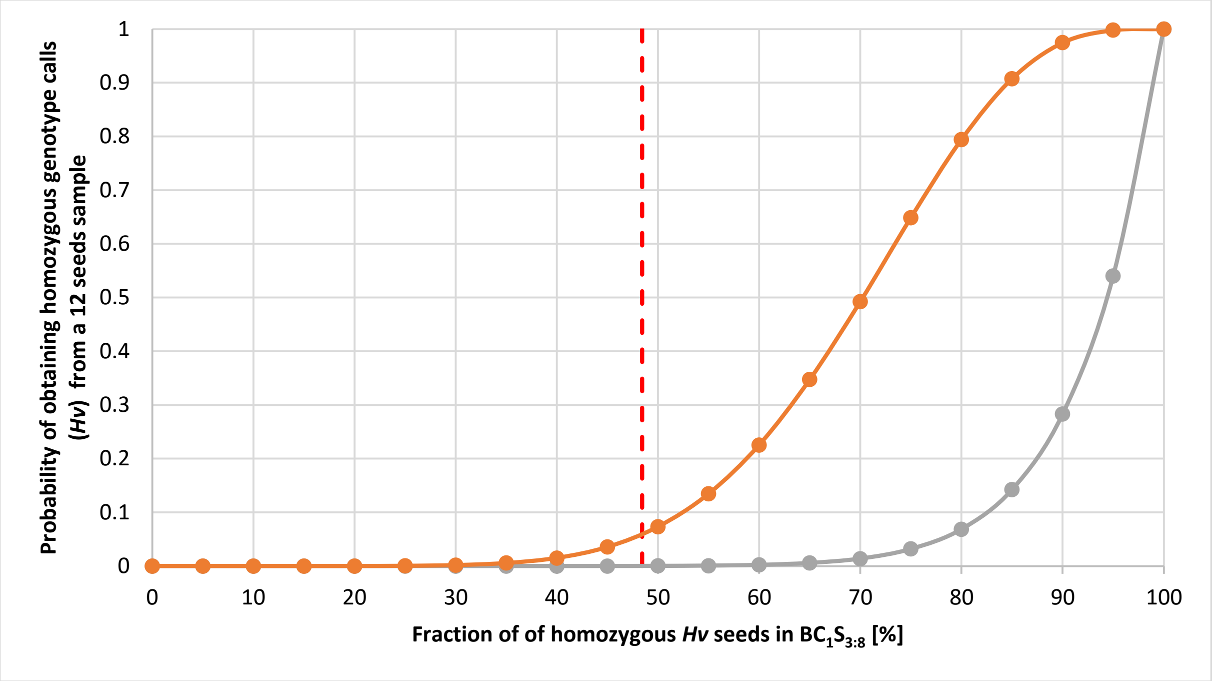

Figure S2. Probability of allele fixation at pooled DNA sampling, exemplified for the *Hv* allele class. Based on the presence of different homozygous seed fractions in $BC_1S_{3:8}$ the probability of obtaining homozygous *Hv* allele calls with the 50k SNP array based on a pooled sample of 12 plants was estimated in binomial trials (Table S3). The red dashed line indicates the expected reference fraction of 0.484375 for homozygous *Hv* alleles in $BC_1S_{3:8}$. Grey and orange lines represent two technically different scenarios of assigning homozygous genotype calls where 12 (grey) or ≥9 (orange) homozygous seeds are required to produce a homozygous signal in Illumina genotyping, respectively. The observed fixation rate of ~0.5 can only be reached when the fraction of homozygous seeds in $BC_1S_{3:8}$ clearly exceeds the expectancy.

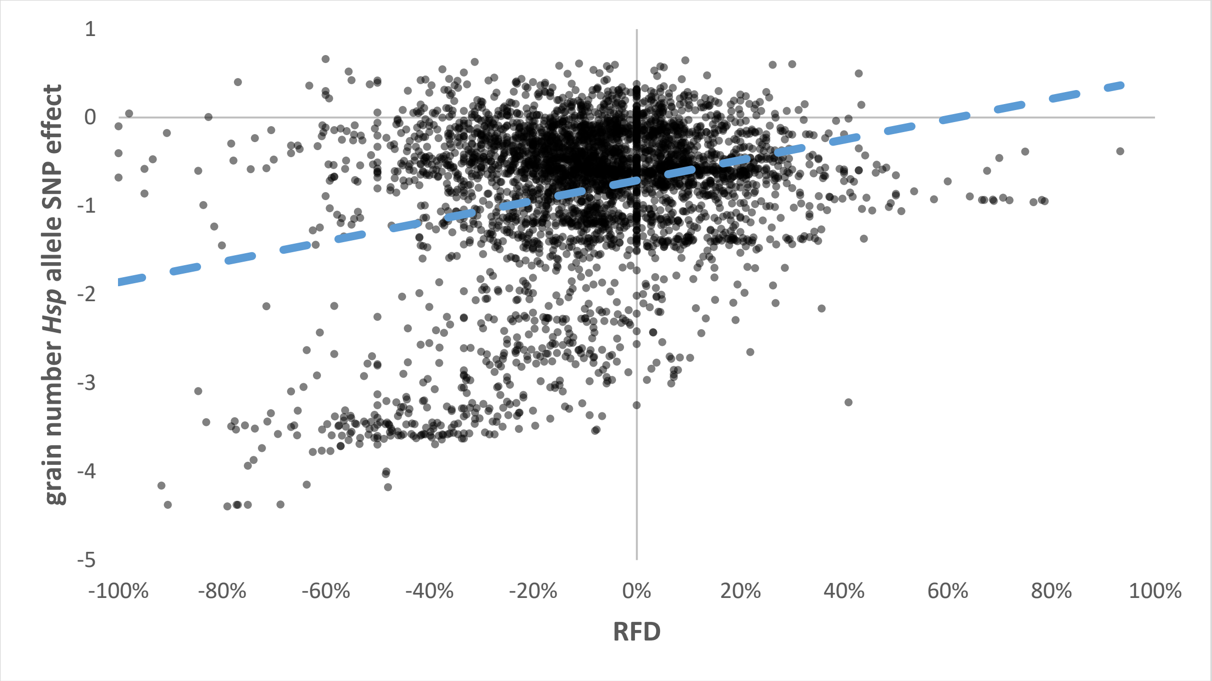

Figure S3. Correlation of estimated wild allele SNP effect for grain number per spike and relative fixation direction (RFD). The Pearson correlation coefficient is equal to *r*=0.28.

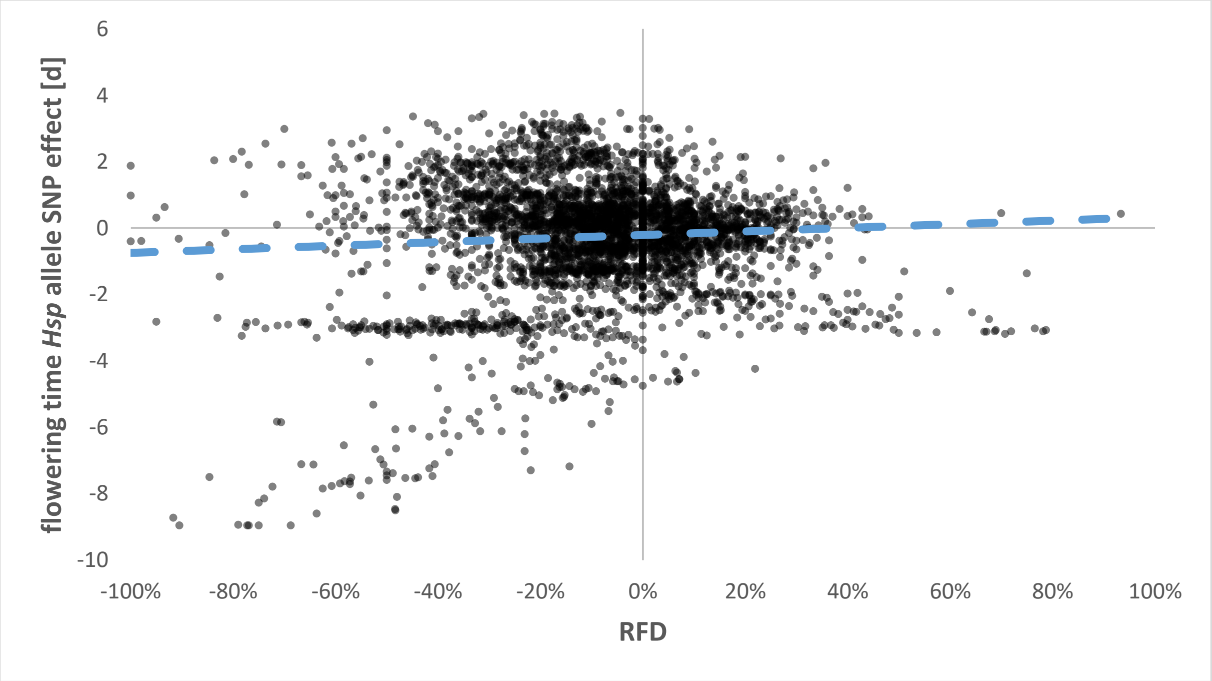

Figure S4. Correlation of estimated wild allele SNP effect for flowering time and relative fixation direction (RFD). The Pearson correlation coefficient is equal to *r*=0.07.

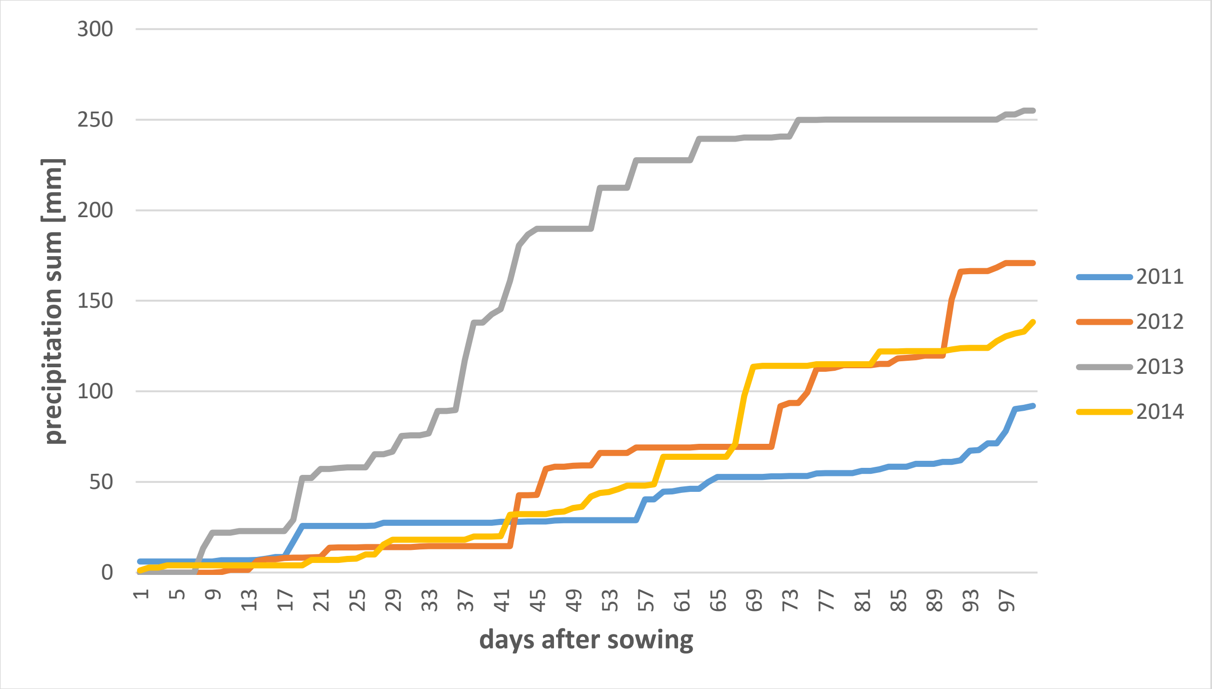

Figure S5. Precipitation sums from sowing until maturity during four seasons of field propagation.
